# How to conceive the dignity of the dead? A dispositional account

**DOI:** 10.1007/s00414-023-02991-6

**Published:** 2023-04-06

**Authors:** Nikolai Münch, Johannes Müller-Salo, Clara-Sophie Schwarz

**Affiliations:** 1 Institute for History, Theory and Ethics of Medicine, Johannes Gutenberg University Medical Center Mainz, Am Pulverturm 13, 55131 Mainz, Germany; 2 Institute of Philosophy, Gottfried Wilhelm Leibniz University Hannover, Hannover, Germany; 3 Institute of Forensic Medicine, Johannes Gutenberg University Medical Center Mainz, Mainz, Germany

**Keywords:** Dignity, Corpse, Posthumous harm, Symbolic value, Moral attitudes

## Abstract

In dealing with human corpses, notions of dignity play a decisive role, especially within legal texts that regulate a corpse’s handling. However, it is quite unclear how the claim “Treat human corpses with dignity!” should be understood and justified. Drawing upon examples and problems from forensic medicine, this paper explores three possible lines of interpreting such demands: (a) positions that closely link the dignity of the human corpse to the dignity of the former living persons and (b) accounts that derive the dignity of the dead from consequentialist considerations. We argue that both lines heavily rely on contestable metaphysical claims and therefore propose an alternative account for the dignity of the dead. Our proposal (c) focuses on action-guiding attitudes and the symbolic value of the dead. Such a conception allows for a variety of morally appropriate groundings of individual attitudes. It avoids metaphysically troublesome premises and, at the same time, allows to classify certain actions and manners of acting as clearly inappropriate and blameworthy.

## Introduction

In dealing with human corpses, notions of dignity play a decisive role. Forensic pathologists, for example, are expected to respect a corpse’s dignity. Some forms of bad practices as handling the corpse’s transportation negligently are criticized as violations of dignity. Such attitudes and moral reactions are well known and intuitively plausible. However, it is quite unclear how the claim “Treat human corpses with dignity!” should be understood and justified. This paper explores three possible lines of interpreting such demands. Throughout the text, we will use examples and problems from the field of forensic medicine to illustrate our arguments.

We will begin by providing some evidence which indicates that “dignity” is the crucial normative notion used in practical and legal contexts to describe those morally relevant attitudes and actions that forensic scientists owe to human corpses (“The notion of dignity within forensic medicine”).

In the following, three conceptualizations of a human corpse’s dignity are analyzed. First, one could link a corpse’s dignity closely to the dignity of the deceased human person or human being to whom that body belonged which has turned into a corpse. Such a conception of a corpse’s dignity depends, as we will argue, on metaphysical claims that are at least controversial and contestable (“Human dignity as source and fundament of a corpse’s dignity”).

Second, it is possible to interpret the claim “Treat human corpses with dignity!” in a broadly consequentialist sense, according to which the corpse’s handling might contribute to a person’s overall welfare. Such a conception, too, needs demanding metaphysical backing, as it relies on the idea that a person’s well-being might be affected through posthumous action (“Dignified handling of corpses based on well-being considerations and rights”).

These two strands of conceptualization are firmly established in the debate. Since they are both problematic, we will, in a third step, present and defend a conception that is focused on action-guiding attitudes. According to this position, to treat a corpse with dignity does neither imply to respect the corpse in itself as a dignified object nor to look at an aggregate value that can be increased or diminished through corpse-related actions. Rather, treating human corpses with dignity implies to take a form of appropriate stance toward the corpse one is handling. Such a conception allows for a variety of morally appropriate groundings of individual attitudes. It avoids metaphysically troublesome premises and, at the same time, allows to classify certain actions and manners of acting as clearly inappropriate and blameworthy (“Adequate attitudes and mutual approval: what it means to handle corpses dignifiedly”).

In the final section, we take a closer look at selected examples of actions that are important for daily routines within forensic medicine. We analyze these examples based on the normative framework developed before and thereby contribute to the professional discussions of standards of best practice (“Applications: dignified handling within forensic medicine”).

As our introductory remarks indicate, we are primarily concerned with mapping the field of discussion and drawing attention to theoretical challenges. Our own systematic proposal can only be sketched out here and certainly requires a more detailed justification.

### The notion of dignity within forensic medicine

Even the examination of biological death, but especially the opening of corpses, was historically a breach of taboo [1] and still is a source of major moral discomfort for many people, albeit autopsies still have a substantial value by enhancing medical knowledge, improving clinical practice and for educational purposes [2, 3]. Therefore, when they perform autopsies, forensic pathologists are professionally involved in what is per se perceived as morally challenging. To be sure, pathologists are given a credit of trust, not only as medical practitioners who are assumed to have a certain professional ethos but also as citizens who, with the help of their education, fulfill a civic duty in the administration of justice, and at the same time as representatives of the community to which they belong. Intuitively, most people are likely to agree with the claim that forensic pathologists can be expected to handle these corpses in ways that do not betray the trust placed on them.^1^

In everyday language, adequate handling of corpses is most often described as “reverent,” “respectful,” or “dignified” handling. The term “dignity” can also be found in several burial laws of various states of the Federal Republic of Germany. The latter regulates, among other things, medical post-mortem examinations, though not court-ordered autopsies in particular. These legal texts refer to the “dignity of the deceased” (for example, the Bavarian Burial Act) [4]. The burial law of North Rhine-Westphalia states that “every woman and man must be in awe of the dead and respect the dignity of the dead” [5]. Schleswig-Holstein has chosen the wording: “The handling of corpses and ashes of deceased persons shall be done with due dignity and respect for the deceased” [6]. Other legal texts are very similar. The burial law of Rhineland-Palatinate and others even takes up the idea that the person who handles the corpse also does so as a representative of his or her community: “The dignity of the dead person and the moral sensibilities of the general public are to be respected.” [7].

The notion of dignity is also a crucial normative concept within relevant forensic medical literature that discusses the appropriate handling of corpses in forensic medicine. For example, a popular German-language textbook states: “A dead person is [...] not equated with a living person, but he or she has a dignity of his or her own.” [8, pp 63-65]. In a survey of forensic medical personnel conducted in 2019, the vast majority of respondents stated that the dignity of the corpse should play a role in the conduct of forensic autopsies [9].

All in all, it becomes clear that particular expectations exist concerning the behavior of people who handle corpses professionally. These expectations are usually linked to the dignity of the deceased. It remains unclear, however, which particular forms of handling respect the dignity of the deceased. This question arises especially in the case of opening corpses, an action that is certainly perceived as a violation of dignity outside of professional contexts.

### Human dignity as source and fundament of a corpse’s dignity

The first and maybe foremost possibility to analyze the claim “Treat human corpses with dignity!” is to assume that the corpse is a bearer of dignity that is derived from human or personal dignity. It is broadly accepted that human beings possess dignity and that, therefore, they are entitled to a whole bundle of fundamental, human, or basic moral rights. It seems promising to base the concept of a corpse’s dignity on such a broader concept of human dignity: Corpses are, after all, the mortal remains of human beings, of persons whose possession of dignity is beyond dispute. In a way, the corpse participates in the person’s dignity and should, as a consequence, be treated with the respect a bearer of dignity deserves.

This is, of course, nothing more than a very general description. A closer analysis depends on the particular understanding of dignity one is prepared to accept. Within the long-lasting philosophical discussions on this topic, at least two understandings of dignity should be distinguished. First, one could claim that individual human beings possess dignity due to the very special status that has been given to the species of *homo sapiens* on earth. This view is particularly influential within the monotheistic, not only Christian traditions of conceptualizing mankind as the image of God. The Hebrew Bible famously states: “And God said, let us make man in our image, after our likeness. […] So God created man in his own image, in the image of God created he him; male and female created he them.” [10]. The *imago-dei* concept had a decisive impact on the history of philosophical thinking as well. In a secularized form, such a conception remains important as it is argued that, for example, human embryos possess dignity simply because they are a member of the species of *homo sapiens*.^2^

Second, dignity could be understood in broadly Kantian terms: Human persons possess those capacities needed for free and autonomous action.^3^ Because of such rational capacities, human beings are *ends in themselves* and should be treated accordingly, as Kant famously states in the *Groundwork of the Metaphysics of Morals*: “I say that the human being and in general every rational being exists as an end in itself, not merely as a means to be used by this or that will at its discretion; instead he must in all his actions, whether directed to himself or also to other rational beings, always be regarded at the same time as an end.” [13, p. 37]. Later on, within the *Metaphysics of Morals*, Kant clarifies the relation between “dignity” and the idea of an “end in itself”: As an end in himself, man “possesses a *dignity* by which he exacts *respect* for himself from all other beings in the world” [14, p. 186]. It is the very special value man possesses because of his being an end in himself that Kant calls “dignity.”

Based on such a conception, it is possible to locate the dignity within the human corpse itself. The corpse itself demands our respect as it is a dignified object. Charles Foster gives an interesting example: “[…] Consider a patient who has given his body to be dissected by medical students. One of the medical students cuts off the patient’s ear and takes it home to be used as an ashtray. Why not? Dignity, again, most would say.” [15, p. 5]. Imagine this patient lived a very lonely life. There might be no mourning relatives; there might be hardly someone who remembers him at all. Consequently, it seems inappropriate to say the student should not cut off the corpse’s ear *because* such behavior would hurt the feelings of those who cared for the deceased. Still, most would share the intuition that the student’s action is immoral and unacceptable and would probably justify a correspondent moral verdict by referring to the dignity the corpse possesses in itself.

Although such a connection between human dignity and a corpse’s dignity seems intuitively plausible, it raises serious philosophical concerns. If human beings possess dignity because of their belonging to the species of *homo sapiens*, one could claim that the human corpse possesses dignity just because it is an entity somehow belonging to *homo sapiens*. Now, even this claim is philosophically contested. In ordinary language, we have no problems in talking about dead persons and dead humans, like in the sentence, “We will bury aunt Mary tomorrow.” However, what is the exact relation between aunt Mary as a former living person and the corpse that now lies in the coffin? What happened to aunt Mary when she died? Did she cease to exist – as a person and/or as a human being? Or does she still exist and persist – again, as a corpse, as a person and/or human being? It has been argued that ordinary language talk about the dead aunt Mary is just an illusion and that there is no such thing as a dead person or a dead human being. According to this view, a person’s and human being’s existence ends at the moment of death [16, p. 27; 17, p. 270]. Dying causes a fundamental ontological change: The human being ceases, and the corpse that is left is not identical with the former living human being; it is a mere thing [16, p. 162; 17, p. 33; 18]. Arguably, there is a “spatio-temporal continuity of some sort” [18, p. 151] between aunt Mary’s body while she was alive and the corpse in the coffin. But this spatio-temporal continuity is not sufficient to claim the persistence of aunt Mary or a human being, for humans are living beings, and living beings have biological criteria for persistence in time: “All of that frenetic, highly organized, and extremely complex biochemical activity that was going on throughout the organism comes to a rather sudden end, and the chemical machinery begins immediately to decay.” [18, pp. 151–152].

This “termination thesis” [16] was rejected by the argument that corpses do share a sufficient number of features with living human bodies to claim a substantial identity between a (living) body and a corpse. For example, both consist of the same organic material. Body and corpse are genetically identical. The corpse resembles the (living) human body externally and as well with regard to its internal organization – that is why corpses can be used in medical education [19, pp. 110-111]. So, if the corpse is identical to the living human body, it also belongs to the species *homo sapiens*. The dispute between the termination thesis’ proponents and its opponents is obviously metaphysical in nature, centered on the problem of an object’s diachronic persistence [20]. We cannot settle this question here, of course. But suppose that humans (at least for some time) continue to exist after their death as corpses and that corpses, thereby, in fact, belong to the species *homo sapiens*. Suppose that all metaphysical difficulties attached to such a position can be overcome. Even then, such a position can be criticized as a clear instance of speciesism as it is well known within animal ethics:^4^ It is one thing to describe a corpse as belonging to a certain species. It is quite another, rather unwarranted step to base some normative claims on such an ontological description [22, pp. 124-128].

The second interpretation of human dignity claims that the human corpse possesses dignity because it is the corpse of a being that possessed dignity-constitutive attributes like rationality or personal autonomy. Such a claim seems plausible only under the condition that the former person or human being is identical or at least connected to the human corpse in a dignity-preserving way. To defend such a claim, one has to accept certain metaphysical propositions concerning the relation between corpse and deceased person, maybe as well concerning the possibility of an afterlife or even with regard to the issue of corporeal resurrection.^5^ Such propositions are surely contestable. Even if one might accept some of them, one nevertheless has good reasons not to base an applied ethics of handling human corpses on worldview-dependent premises. Applied ethics that guides the professional behavior of, for example, forensic physicians who work in a pluralistic society should be acceptable regardless of individual faith or metaphysical conviction.

We do not claim that it is impossible to solve the problems we identified. Our claim is rather modest: These objections show that the concept of a human corpse’s dignity cannot simply be connected to personal or species-related dignity without thereby accepting a variety of metaphysical and ethical assumptions that are at least controversial. Therefore, it seems promising to look for other interpretations of treating corpses with dignity if one wishes to develop an applied ethics of handling human corpses that is acceptable independently of specific metaphysical, cultural, or religious backgrounds.^6^

## Dignified handling of corpses based on well-being considerations and rights

Besides species-related or Kantian approaches, there have been attempts to base moral duties toward human corpses on consequentialist accounts. Charles Foster, for example, argues for an approach to human dignity, which he understands as “facilitating the full humanness of each person: of encouraging flourishing” [15, p. 6]. And he thinks that it is possible to speak of the flourishing of the dead [15, pp. 7-8]. But this is not obvious. What could it mean for a dead person to flourish? The former living human person is dead. There is nobody who has feelings, emotions, or experiences anymore. In what way could anybody be better or worse off by something that happens after her death? Part of Foster’s answer is this: A person wishes to survive her death.^7^ Disregarding a surviving wish impairs her flourishing. Critics, of course, would claim that there is no subject that could have wishes and could flourish because her wishes are respected.

In a debate concerning the possibility of posthumous harm, this objection has been coined “the problem of the subject” [26, p. 185] [27, 28]. A common response is the so-called “Feinberg-Pitcher Argument” [28, p. 13; 29, p. 173]. To solve the problem of the subject, they argued, you have to differentiate two ways in talking about a dead person: You can describe her as a post-mortem person – as she is now “mouldering, perhaps, in a grave” [30, p. 184] – or as the person she was in her life, that is as an ante-mortem person. Now, while the post-mortem person cannot be harmed, the ante-mortem person can. “Consider Mrs. White, for instance. Mrs. White, remember, was very proud of the business that she had established. We may assume that she had a strong desire that it should survive for a long time after her death as a kind of monument to her industry and skill. This desire is defeated when the business collapses soon after her death. I maintain that the wrecking of her business thus harms Mrs. White – the living (ante-mortem) Mrs. White – even though it occurs when she is dead.” [30, p. 184].

The ante-mortem person as an actual subject of harm is related to the state of affairs after her death if her interests, wishes, or desires are directed during her lifetime at certain post-mortem events. This seems to avoid the problem of the missing subject. It does so, however, at a high cost, “for something to happen after a person’s death that harms the living person he was before he died” [30], which seems to implicate an implausible kind of backward causation [30, 31]. How could an event possibly change the past and the well-being of somebody that has passed away? Proponents of the Feinberg-Pitcher line of argument insist that “posthumous harms do not entail backward causation because they too do not entail physical causation at all” [31, p. 92]. Instead, the idea is that “the significance of acts and enterprises is often determined by things that happen at a later date” [29, p. 173]. So, in the case of Mrs. White, the post-mortem collapse of her business does not cause harm to her ante-mortem. Rather, with the collapse, “it becomes apparent to us for the first time that it was true all along” [31, p. 92] that Mrs. White’s efforts were futile and that her interests were harmed, though she didn’t know it. After identifying a proper subject of harm – the ante-mortem-person – the argument tries to locate the timing of harm within the subject’s lifetime, although the relevant knowledge concerning such harm is available only post-mortem.

The “Feinberg-Pitcher account of how posthumous harm is possible […] is dominant in both discussions on the metaphysics of death and hence in the bioethical literature that draws on it” [28, p. 6]. But there are forceful critics, too. Regularly, they remain skeptical with regard to Feinberg and Pitcher’s second argument that tries to avoid implausible backward causation. While it is true that some properties could be ascribed to persons depending on events occurring after their death, this holds not for harm these critics argue: If person A shoots another person B who shoots back with the result that A dies five minutes later, the wounded persons B survives a day longer and then dies as a result of her gunshot injury, then the death of B (that is a posthumous event, occurring after A’s death) makes it true that A was a killer [32, p. 336]. But the property of being harmed cannot be described analogously. Being harmed by thwarting someone’s interests (as Feinberg and Pitcher understand harm) presupposes that the person in question has the capacity to be affected somehow, even if such affection is indirectly and almost unlikely [33, 34]. So, the analogy drawn with the help of the cited killer example or similar cases might be misleading [28, pp. 14-15]. The Feinberg-Pitcher defendants, in turn, point to cases where we are inclined to say that a living person can be harmed without the possibility of being affected by the harming act: Suppose in the above-cited example of Mrs. White that her business collapsed shortly before her death, but “her friends, eager to save her from disappointment, conceal or misrepresent the facts. She dies contented.” [31, p. 89]. In that case, Feinberg argues, “[i]t would not be very controversial to say that the woman […] had suffered grievous harm to her interests although she never learned the bad.” [31, p. 89]. If Mrs. White suffered harm in this case and if the fact that she never knew about the setback of her interests is irrelevant, then it is hard to claim that her death makes a difference [26, p. 187; 35, p. 495].

Other authors developed similar cases that try to show that we intuitively do accept unaffecting harms regarding the living and therefore should do so also regarding the dead [35, 36, pp. 4-5; 37, pp. 193-195]. But even if you drop the presupposition that the notion of harm comes with an “experience requirement” [26, p. 187], it still remains unclear which criteria must be met to ascribe “harm” retroactively. Whether you accept the claim that such a posthumously ascription of harm is, in principle, possible will depend inter alia on your account of harm and well-being [38, 39, pp. 125-127]. We cannot discuss these fundamental issues here. In any case, the discussion about posthumous harm is based on the forceful intuition that there are acts and events regarding the dead that are morally doubtful – and that these doubts have something to do with the ante-mortem person. Even if one shares these intuitions, there might be other explanations for them than to draw a parallel between harming the living and harming the dead, as the difference between life and death surely is of crucial moral importance. In important ways, the dead are beyond harm for there does no longer exist a living human being with interests, emotions, or feelings [34]. So, if one could harm the corpse, it is another kind of harm or setback of interests that might be at stake.^8^

An alternative account that could cover the intuitions about the possibility of harm in the context of dealing with corpses is this: Not the dead are harmed; it is the living that is harmed. This harm might occur directly via harming the surviving dependents of the dead or more abstractly, for example, in some rule-consequentialist fashion according to which respecting the wishes or interests of the dead “is likely to avoid adversely affecting the well-being of living persons who want their own future wishes honoured” [27, pp. 311-322; 34]. Such claims might be correct. Nevertheless, there seems to be a strong intuition that there are not only considerations regarding the living that govern our treatment of corpses and dead persons but also the corpse and/or the ante-mortem person that matters morally. Is it possible to spell out this intuition without relying on precarious analogies concerning posthumous harm or metaphysical convictions?

In our view, one promising idea is this: In treating a human corpse inappropriately, a person reveals an attitude of dishonoring and disrespecting human dignity in a symbolic sense. To illustrate this, take an example discussed within the philosophy of violence: There is a huge difference between throwing some stones at a tool shed’s window and throwing some stones at a synagogue’s, a mosque’s, or a church’s window. Seen from the outside, both actions might appear to be very similar. Nevertheless, whereas the first might be described as some form of damaging or destruction, the second might be described as an act of violence. Why is there such a difference? The answer is easy: A house of prayer has a high symbolic value. Damaging such a place implies attacking the dignity of those attached to it. This holds true even if there is no individual person that is hurt and whose dignity is affected directly.^9^

An attack on an object of high symbolic value reveals an attitude of disrespect for its value. The same line of argument can be used in addressing the human corpse which surely is an object of high symbolic value in a twofold sense. First, the corpse obviously represents and, therefore, symbolizes the deceased person to whom the body belonged. Her dignity can be violated symbolically through the corpse’s mistreatment. Political history is full of cases where a dead enemy’s body was mistreated in order to humiliate the dead person. Just take the case of Cicero being murdered and his corpse being publicly mutilated and displayed at the *Roman Forum*. And as Michael Rosen remarks, “one of the features that characterized many of the most violent and destructive acts of the twentieth century was the humiliation and symbolic degradation of the victims” [47, p. 158]. As in the case of damaging a religious building, the significance of such an act goes beyond the material damage done. The violation here – at least in parts – comes from the expression of disrespect.

Second, the idea of human dignity itself might be symbolized through a human corpse and, consequently, violated through a corpse’s mistreatment. What counts as disrespectful handling will thereby depend on cultural contexts. Take the example of funereal practices: Obviously, different cultures developed manifold rites and traditions of mourning and burying. If a person gets to know another culture’s funereal practices, she might learn that respect toward a deceased person’s dignity (and maybe human dignity in general, at least up to a certain degree) can be shown in very different ways through actions totally unfamiliar to her. This observation contains an important insight: One does not only have to look at concrete actions if one asks whether a corpse is handled dignifiedly. Rather, one has to analyze the action-guiding attitude: Do the persons involved understand their own actions as dignified handling? Do they intend to treat the corpse with respect? Unlike in the case of violations of the dignity of living persons, disrespect for the dignity of the dead concerns mainly the expressive and symbolic level. While torturing or restricting freedom of speech is a material violation of a living person’s dignity, the example of highly varying funeral rituals shows that with the dead, the material aspects take the back seat. Here, the symbolic level gets important, stressing the underlying attitude that can be expressed by varying material acts depending on cultural contexts.

### Adequate attitudes and mutual approval: what it means to handle corpses dignifiedly

As both interpretations of the demand: “Treat human corpses with dignity!” that we have analyzed so far face significant difficulties, it seems worthwhile to develop an account that rests on different grounds. It is neither connected to treating the human corpse as a dignified object nor is it associated with the duties of contributing to a person’s overall (even posthumous) welfare. As indicated in the previous section, this third account rather focuses on attitudes that those persons who handle the corpse develop toward it.

To begin with, an attitude can be broadly described as an evaluative stance a person takes toward another person, an object, an action, or a state of affairs. An attitude is based on experience and on certain normative grounds like moral convictions, religious and legal norms, or axiological propositions. A person’s experience with a certain entity is evaluated by relying on those normative grounds the person accepts or has access to. “Experience” should be taken in a broad sense, including not only all forms of perception and sensual experience but also testimonial experience and maybe even imagination as well. As well, “evaluation” should not be understood as a process of which the person is always aware of: It might occur spontaneously, or it might be partly unconscious and automatic.

As a consequence of experience and evaluation, a person might form an attitude toward the experienced entity. To take a simple example, A person buys a second-hand car. Shortly after buying the car, the person’s car mechanic finds out that the car is in need of expensive reparations the car salesman did not mention to her. Based on some normative grounds the person shares – for example, the moral demand “if selling something, you should tell the truth about its current conditions” – she evaluates the salesman’s behavior as an instance of cheating and lying. This might cause the development of an attitude of distrust and doubt toward salesmen in general that influences her behavior when negotiating future sales agreements.

Not every personal attitude must influence our behavior decisively. One can notice that one dislikes a relative and can realize at the same time that this attitude is not well-grounded. When knowing this, one can actively try to diminish the attitude’s influence on one’s own behavior.

Based on such an understanding of attitudes, it is possible to give a third answer to our main question: A person treats a human corpse with dignity if and only if she develops an attitude toward the corpse that is based on adequate normative grounds and that adequately guides her behavior when handling the corpse or interacting with it.

The plausibility of such an account certainly depends on giving sense to “adequate normative grounds” and “adequate behavior guiding.” We begin with the second point, the notion of an attitude that adequately guides a person’s behavior. “Behavior” contains at least two things, a person’s actions and her manner of acting. In many contexts, the manner of acting is morally relevant as it often reveals a person’s attitude.^10^ One might do the right, appropriate things; nevertheless, one might be criticized if one performs such actions in a problematic way. A forensic pathologists’ actions during an autopsy might be technically fully adequate and might still be problematic as these actions might be performed disrespectfully, harshly, or hasty.^11^

An attitude can only be an adequate guide if it, in fact, influences a person’s actions or manners of acting. When the attitude guided the action, the question “Why did you do X and why did you do X in the way you did?” cannot be answered satisfactorily without mentioning the attitude. There is no reason to address issues within action theory here. Nevertheless, it becomes clear that an attitude cannot be described as action-guiding if it plays no role in explaining the course and grounds for acting.

Furthermore, an action is only adequately guided by an attitude if it reflects the attitude’s content. Handling a corpse harshly, brutally, or negligently is incompatible with an attitude of respect and, therefore, cannot reflect such an attitude. If a person claims that she possesses an attitude of respect with regard to human corpses and, nevertheless, handles a corpse disrespectfully, several things might have gone wrong. She might simply be a liar and might claim to hold an attitude that she does not possess. She might be mistaken with regard to her own attitudes. She might lack practical knowledge and might not be aware of the fact that certain actions and manners of acting do not reflect an attitude of respect. She might, in principle, possess an attitude of respect. Due to specific circumstances, however, this attitude does not guide her behavior: She might simply have a bad day. She might be absorbed in her own thoughts. As this shows, there are many possibilities to explain why an action does not reflect a certain attitude, even in cases where a person plausibly claims to possess such an attitude.

Given these further explanations, it should be clear what it means for an attitude to guide a person’s behavior adequately. But what does it mean to have “adequate normative grounds” for an attitude? In the “Human dignity as source and fundament of a corpse’s dignity” section and the “Dignified handling of corpses based on well-being considerations and rights” section, we have examined two conceptions of a corpse’s dignified handling that can be interpreted as attempts to describe and defend normative grounds. Certain beliefs about posthumous harm or religious faith in the resurrection and eternal afterlife might provide normative grounds for a person to develop an attitude of respect toward a human corpse. It became clear, however, that such normative groundings raise serious problems. Even if one thinks that some of these problems can be overcome, it is beyond doubt that these grounds remain highly controversial. Therefore, we propose another understanding of “adequate normative grounds,” that is, in a certain sense, neutral with regard to content. We do not claim that specific normative grounds are adequate. Rather, we propose a procedural account: A normative ground for an attitude is adequate if and only if it is intersubjectively comprehensible and approvable from a practice-oriented point of view.

What does this mean? Take the following example: A police officer attending an autopsy gets the impression that the forensic pathologist and her team handle the corpse in front of them with great care and respect. Our police officer asks the physician why she is executing the autopsy in this particular way. The pathologist answers that she is a devoted Christian and believes in corporeal resurrection and an eternal afterlife. Our police officer might be an atheist and might firmly believe that such religious faith is inconsistent, maybe even absurd. Nevertheless, he can acknowledge that her faith gives her a normative ground to handle the corpse with care. In this sense, the physician’s normative ground is intersubjectively comprehensible.

The conception outlined here allows for mutual comprehension in cases where it is impossible to agree on the truth or correctness of specific normative grounds. Therefore, this position avoids any strong metaphysical claim. Rather, it highlights that an adequate attitude with regard to human corpses might rest upon very different normative grounds. To give just a few examples, religious faith, as described in the example, might be a comprehensible ground. As well, the relevant attitude might be grounded in an interpretation of one’s own professional duties and might be connected to a professional self-image or a particular perception of one’s own professional role. Feelings of compassion toward the deceased person and her relatives might be another ground. In all these cases, the attitude can be intersubjectively comprehensible, even if other persons do not share the grounds in question.

Intersubjective comprehension, however, is not enough. As indicated above, “adequate normative grounds” are, at the same time, intersubjectively comprehensible *and* approvable from a practice-oriented point of view. What does this mean? It certainly does not mean that a person has to accept another person’s normative grounds and their specific contents. Our atheist police officer will surely not accept the pathologist’s religious convictions. Nevertheless, he can approve of her normative grounds in a practical sense as they lead to an attitude that he considers to be morally acceptable or even virtuous. He can acknowledge that such grounds are adequate as they lead to an appropriate attitude.

This theory surely expresses a primacy of practice. In most cases, we do not ask whether we share the reasons of others for their actions but whether we can approve of their actions. Pluralistic societies are often forced to agree on certain courses of action, even if the participants’ grounds for action may be very different. Therefore, this understanding of a corpse’s dignified handling is certainly based on something like an overlapping consensus as Rawls described it [48, at Part Two, Lecture IV]: It is possible to intersubjectively agree on the adequateness of certain attitudes even though the normative grounds for such an agreement might differ significantly from person to person.

One might suspect that the theory outlined here does not provide enough practical guidance as it does not identify certain actions as instances of dignified or undignified treatment but rather develops procedural criteria of intersubjective comprehension and approval. We believe that such criticism is unwarranted. Remember the example of varying burying practices: One can recognize that certain actions express an attitude of respect and, consequently, instances of dignified handling, even if one operates within a very different cultural code and, as a consequence, would do other things to express one’s attitude of respect.

Moreover, there are certain attitudes toward human corpses that cannot be intersubjectively approved. This is, for example, the case if the human corpse is seen merely as an object one has to work with in order to earn money or to promote one’s own academic career. Relying on such normative grounds, pathologists might develop an attitude of disrespect that surely would not find the approval of, for example, the deceased person’s relatives or some uninvolved spectators. Therefore, the position outlined here can justify moral criticism of attitudes and, as a consequence, of particular actions and manners of acting. At the same time, it avoids the introduction of burdensome metaphysical assumptions competing theories are built upon. As this shows, our proposal is in line with important strands of thought within biomedical ethics that avoid any form of metaphysical groundwork.

Finally, it should be noticed that the proposal outlined here avoids any problems of circularity. Other theories, which tie the corpse’s dignity to the dignity of the human person, the dignity of the human species, or the dignity of the divine creature, often only provide some general advice for practice: Corpses possess dignity; therefore, treat them with dignity! By contrast, our account, as being focussed on a procedural criterion, could guide further developments of best-practice standards. For it holds that we should consider all those different actions as instances of dignified handling of corpses that are intersubjectively comprehensible and approvable from a practice-oriented point of view.

## Applications: dignified handling within forensic medicine

Concrete examples from forensic medical practice can be taken from the aforementioned survey by Schwarz et al. [9]. Forensic physicians and autopsy assistants practicing in Germany were asked in 2019 to participate in an online questionnaire. The answers to the single- and multiple-choice questions were transferred to pivot tables and analyzed descriptively [9]. In addition, the comments entered as free text also provide valuable information on possibly problematic actions in the everyday work of forensic medicine [9]. Factors that cannot be directly influenced as well as those that can in principle be influenced by the protagonists were named as disruptive factors in the dignified handling of corpses. Among the factors that could not be influenced or could only be influenced to a limited extent, room conditions played a role and were criticized by the interviewees as inappropriate. If corpses are stored in an unkempt ambiance, this does not reflect an appropriately respectful attitude in dealing with corpses according to the perception of the respondents. Respondents find themselves in a situation where, by storing corpses in unkempt premises, they are acting contrary to their attitude. Likewise, the behavior of third parties during the delivery and collection of corpses (undertakers) and in the dissection room (colleagues, students, police officers) can only be influenced by the respondents to a limited extent. This refers to rough handling of the corpse, where, for example, the head noisily hits the dissection table when the corpse is being moved. This action by a third party obviously meets with resistance from the interviewees because it cannot be reconciled with a respectful attitude according to the perception of the interviewees. The same applies to bystanders (students, police officers) who, for example, transmit that autopsy participation has primarily entertainment value for them. Viewing corpses as objects of entertainment is an attitude that surely does not rest on the grounds that they are intersubjectively comprehensible and approvable. Even if the behavior does not seem directly inappropriate on the surface, the underlying attitude meets with strong disapproval.

Some respondents mentioned attitudes and actions of the forensic medical staff themselves that they felt were not compatible with dignified handling. For example, actions on corpses were criticized that are based on personal ambition in a scientific career or greed for money since institutes of forensic medicine also seek to work cost-covering. Only a few participants in the study considered autopsies performed for the right reasons and *lege artis* to be problematic per se. However, special actions and circumstances were mentioned in which well-grounded, and professionally correct work was exceptionally perceived as incompatible with the dignified handling of corpses. These include elaborate dissections of, for example, the face in the case of special questions and the autopsies of corpses that are severely decomposed or overweight. On closer examination, however, the question arises as to whether it is not actually the way in which the aforementioned actions are carried out that should be criticized here. In principle, it is technically possible to reconstruct the face after elaborative dissections. Here, it is more likely that a hasty method of working, for example, in the case of a heavy workload and time pressure, is problematic. The procedure for the autopsy of severely decomposed or overweight corpses basically corresponds to the usual procedure. Here, the question arises whether it is not rather the way of speaking about these circumstances, for example, the expression of disgust, that is to be identified as a fault in the dignified handling of corpses. Overall, the way in which actions are performed is perceived as problematic much more often than the actions themselves. Here, the respondents of the questionnaire were mainly concerned with clean work (which includes, for example, frequent rinsing away of leaked body fluids), quiet, careful work, and cosmetically impeccable closing of the corpse after the autopsy. In our view, this sensitivity toward manners of acting hints at the importance of attitudes in evaluating the handling of corpses. After all, a person’s attitude is often revealed not only in the action itself but also in the way it is performed.

It should be noted, though, that not all legal regulations regarding the treatment of human corpses are spelled out explicitly in the language of dignity. For example, in the German penal code (§168 StGB), the “disturbance of peace of death” is a punishable offense. Such disturbances are, for example, the unauthorized taking away of corpses, the damaging or defaming of burial sites, or acts that express a special disrespect against the deceased [49]. Expressing disrespect is arguably itself – at least in parts – not only a question of the material action taken but also ultimately a question of the underlying attitude. This explains why the intrusion in the physical integrity of a body is not always per se perceived as a moral (or legal) problem – be it during an autopsy or by certain burial forms like cremation because such acts can reasonably be understood as guided by attitudes based on adequate normative grounds.

## Conclusion

Empirical evidence, as has become clear in the previous section, provides hints that attitudes are of central moral importance in the respectful treatment of human corpses. Attitudes, which can be expressed, for example, in the manner of acting, should therefore be at the center of a theory of the dignified treatment of corpses. In this paper, the main features of such a theory have been outlined. It goes without saying that these require in-depth discussion and justification. It has become clear, however, that an attitude-oriented theory is not confronted with those metaphysical challenges that other conceptualizations of a human corpse’s dignity face. In a pluralistic world, a theory that does not depend on concrete answers to ultimate questions is undoubtedly at an advantage when it comes to questions of practical applicability.

## Data Availability

Not applicable.

## Footnotes

1 Increasingly image-based autopsies are used as alternatives to traditional autopsies, and at least in the perception of some people, these less intrusive autopsies are morally also less troublesome than traditional autopsies [3]. As will get clear in the course of our argumentation, the dignified handling of bodies does from our point of view not per se call for the least intrusive measure as the material integrity of the corpse is not the only nor the decisive moral aspect of a dignified handling, as our understanding is disposition based.

2 Such an understanding of a human embryo’s dignity has been, to mention an important example, decisive for the German legislation on embryonic research or on techniques of genetic diagnosis like the PGD. For the German legal development [11, 12].

3 The important question for Kantian ethics, whether and why human beings who are not persons can possess dignity, is irrelevant to our discussion and will therefore not be pursued further here.

4 Peter Singer has famously developed the notion of speciesism [21, pp. 73-86].

5 For a philosophical defence of the (Christian) doctrines of corporal resurrection and “temporary disembodiment” between the moment of death and the moment of resurrection [23].

6 For a current introduction into the philosophy of death that discusses those metaphysical questions, we mentioned in this section alongside with ethical issues regarding the human dead [24].

7 Foster gives four other arguments why flourishing could posthumously be impaired. See further reflections on “asynchronic wishes” that might be applied to the case of a deceased person’s wishes as well [25].

8 Some authors argue that an interest-based account of well-being is not able to cover posthumous harm while an account of harm based on the achievement of some goals might cover the intuitions behind the possibility of posthumous harm [40–42] But these adaptations of the Feinberg-Pitcher account do not rebutt the objection that the dead are beyond harm for there is nobody anymore that cares about those important goals or life-projects.

9 See further, e. g., David Parkin, who differentiates between violence as physical destruction and violence as metaphysical desecration. [43, p. 205]. Cf. the influential account of E. Canetti [44, p. 16]. For a critical analysis of whether and in which sense inanimate things can become objects (or even “victims”) of violence [45, 46, pp. 80-84].

10 For an informative example, see the ICRC’s “Guiding Principles for the Dignified Management of the Dead in humanitarian emergencies and to prevent them becoming missing persons” online at: https://shop.icrc.org/guiding-principles-for-dignified-management-of-the-dead-in-humanitarian-emergencies-and-to-prevent-them-becoming-missing-persons-pdf-en.html, last access: 18.01.2023. Several principles included in this document can be interpreted as putting special emphasis on manners of acting. Thus, for example, it is stated that “final disposition of dead persons should be undertaken in a manner that respects their dignity and privacy, and that of their family members and communities” (principle 19). Furthermore, the need to respect religious, cultural and local beliefs, costumes, and traditions, which is often a question of manner, is emphasized (e.g., principles 12, 18).

11 For the importance of questions of manner within forensic medicine, see [9]; for an ethical account on the importance of manners of action see [50].
